# Association of Chronic Hyperglycemia and Glycemic Variability with Mortality in COVID-19: Meta-Analysis of Cohort Studies

**DOI:** 10.3390/medicina62020310

**Published:** 2026-02-02

**Authors:** Ana-Maria Pah, Dragos-Mihai Gavrilescu, Diana-Maria Mateescu, Ioana-Georgiana Cotet, Maria-Laura Craciun, Eduard Florescu, Simina Crisan, Adina Avram

**Affiliations:** 1 Cardiology Department, “Victor Babes” University of Medicine and Pharmacy, Eftimie Murgu Square 2, 300041 Timişoara, Romania; anamaria.pah@umft.ro (A.-M.P.);; 2 Department of Orthodontics, Dental District, Strada Zăgazului Nr. 3, ONE Floreasca Vista, Sector 1, 014261 Bucharest, Romania; 3 Doctoral School, Department of General Medicine, “Victor Babes” University of Medicine and Pharmacy, Eftimie Murgu Square 2, 300041 Timisoara, Romania; 4 Department of Infectious Diseases, “Victor Babes” Infectious Diseases and Pulmonology Clinical Hospital, 13 Gheorghe Adam Street, 300310 Timisoara, Romania; 5 Department of Internal Medicine I, “Victor Babes” University of Medicine and Pharmacy, Eftimie Murgu Square 2, 300041 Timisoara, Romania

**Keywords:** COVID-19, glycemic control, glycemic variability, hyperglycemia, HbA1c, ICU outcomes, mortality, meta-analysis, SARS-CoV-2

## Abstract

*Background and Objectives*: Dysglycemia is a major determinant of adverse outcomes in COVID-19, yet the separate contributions of poor glycemic control and glycemic variability (GV) remain incompletely defined. We conducted a systematic review and meta-analysis of observational cohort studies (both prospective and retrospective) to quantify the impact of chronic hyperglycemia and glucose instability on disease severity, intensive care requirements, and mortality in patients with COVID-19. *Materials and Methods*: We searched PubMed, Scopus, and Web of Science from January 2020 to October 2024 for observational cohort studies reporting clinically relevant COVID-19 outcomes stratified by glycemic control or GV. Dysglycemia definitions varied across studies (HbA1c-based chronic hyperglycemia, fasting glucose, or admission/in-hospital hyperglycemia). GV was assessed using metrics including mean amplitude of glycemic excursions (MAGE), standard deviation (SD), coefficient of variation (CV), or maximum daily glucose difference. Twelve studies met inclusion criteria and were included in qualitative synthesis; five studies were eligible for quantitative synthesis of clinical outcomes. Random-effects DerSimonian–Laird models were applied due to anticipated clinical heterogeneity. Heterogeneity was evaluated using Cochran’s Q, τ^2^, and I^2^ statistics. *Results*: Overall, 12 observational studies (9 prospective and 3 retrospective cohorts; *n* = 1,008,310 patients) were included. In quantitative analyses of five eligible cohorts, poor glycemic control was associated with a significantly increased risk of severe or critical COVID-19 (pooled RR = 1.75, 95% CI: 1.45–2.11; I^2^ = 29%), ICU admission (RR = 1.54, 95% CI: 1.18–2.01), and mechanical ventilation (RR = 1.72, 95% CI: 1.31–2.26). Three studies evaluating GV demonstrated a strong association with adverse outcomes (pooled RR = 2.07, 95% CI: 1.71–2.50; I^2^ = 0%); this low heterogeneity should be interpreted cautiously given the limited number of studies. GV remained associated with mortality in multivariable models, indicating that glycemic variability is separately associated with mortality as a clinically relevant prognostic risk marker in hospitalized COVID-19 patients. *Conclusions:* Both chronic hyperglycemia and elevated glycemic variability are each associated with increased risk of severe COVID-19 outcomes. Glycemic variability appeared to be a consistent, low-heterogeneity prognostic marker of mortality, being separately associated with higher death risk in hospitalized COVID-19 patients, highlighting its potential utility as a dynamic metabolic biomarker. Early identification and targeted management of dysglycemia—especially glucose instability—may improve prognosis in hospitalized COVID-19 patients. PROSPERO: CRD420251250718.

## 1. Introduction

The coronavirus disease 2019 (COVID-19), caused by severe acute respiratory syndrome coronavirus 2 (SARS-CoV-2), has disproportionately affected individuals with pre-existing metabolic disorders. Among these, type 2 diabetes mellitus (T2DM) has consistently been associated with higher risk of severe or fatal COVID-19, being associated with higher rates of acute respiratory distress syndrome, multiorgan dysfunction, and mortality [1,2,3]. This excess risk extends beyond epidemiological association and reflects a complex interaction between viral pathogenesis, immune dysregulation, and metabolic instability.

Dysglycemia—whether chronic, stress-induced, or fluctuating—profoundly alters host defense mechanisms. Hyperglycemia impairs neutrophil chemotaxis, reduces complement activity, disrupts macrophage phagocytosis, and attenuates T-cell responses, while simultaneously promoting oxidative stress, excessive cytokine release, and endothelial dysfunction [4,5,6,7]. These changes facilitate viral propagation, amplify pulmonary and systemic inflammation, and aggravate the thrombo-inflammatory complications characteristic of severe COVID-19 [8]. Conversely, SARS-CoV-2 infection can itself destabilize glucose homeostasis through cytokine-driven insulin resistance, counter-regulatory hormone release, and possible β-cell injury, thereby establishing a bidirectional metabolic–inflammatory cycle [9,10,11]. In this context, chronic hyperglycemia reflects pre-morbid glycemic burden (HbA1c), whereas stress hyperglycemia reflects acute illness–related glucose elevation at admission or during hospitalization. In this review, “chronic hyperglycemia” refers exclusively to HbA1c-based glycemic burden, while ‘stress hyperglycemia’ denotes admission or in-hospital glucose elevations captured by fasting or random glucose measurements.

Therapeutic factors further complicate glycemic control during COVID-19. Glucocorticoids—the standard of care for moderate-to-severe disease—frequently cause marked hyperglycemia and glycemic variability, necessitating intensive metabolic monitoring [12]. Emerging evidence also suggests that SARS-CoV-2 may precipitate new-onset diabetes through β-cell stress, immune activation, and microvascular injury, leading to persistent post-acute metabolic impairment [13].

However, the available evidence remains fragmented. Prior reviews have frequently relied on predominantly retrospective datasets, heterogeneous definitions of dysglycemia, inconsistent adjustment for confounders, and limited systematic evaluation of in-hospital glycemic variability as a dynamic prognostic marker [14,15]. As a result, the separate contributions of chronic hyperglycemia, stress hyperglycemia, and glucose instability to critical illness and mortality in COVID-19 remain incompletely quantified.

To address these gaps, we conducted a systematic review and meta-analysis of observational cohort studies evaluating the associations of chronic hyperglycemia (HbA1c), admission hyperglycemia, in-hospital glycemic variability, steroid-induced dysglycemia, and new-onset diabetes with clinically relevant COVID-19 outcomes. Specifically, we aimed to evaluate associations with (i) all-cause mortality, (ii) ICU admission, (iii) need for mechanical ventilation, and (iv) severe/critical COVID-19, in order to inform evidence-based glycemic monitoring and management in hospitalized patients with COVID-19.

## 2. Materials and Methods

### 2.1. Protocol Registration and Reporting Standards

This systematic review and meta-analysis were conducted following the Preferred Reporting Items for Systematic Reviews and Meta-Analyses (PRISMA) 2020 guidelines [16] and the methodological recommendations of the Cochrane Handbook for Systematic Reviews of Interventions.

The study protocol was prospectively registered in the International Prospective Register of Systematic Reviews (PROSPERO) under registration ID CRD420251250718, prior to data extraction and analysis. All amendments to the protocol were logged and updated in the PROSPERO record. A completed PRISMA 2020 checklist is provided in Supplementary Table S1.

### 2.2. PICO Framework

The research question was defined using the PICO structure to ensure methodological transparency and reproducibility: Population (P): Adults (≥18 years) with SARS-CoV-2 infection/COVID-19, with established type 2 diabetes mellitus (T2DM) or dysglycemia (including new-onset dysglycemia during acute infection). Exposure (E): Chronic hyperglycemia (HbA1c), admission hyperglycemia (admission glucose), in-hospital glycemic variability metrics (e.g., SD, CV, MAGE, maximum daily glucose difference), steroid-induced hyperglycemia/dysglycemia, and new-onset diabetes. Comparator (C): Good vs. poor glycemic control; normoglycemia vs. hyperglycemia; low vs. high glycemic variability. Outcomes (O): All-cause mortality, ICU admission, need for mechanical ventilation, and severe/critical COVID-19. The primary mortality endpoint for quantitative synthesis was in-hospital or ICU mortality, with 28-day mortality considered in sensitivity analyses where reported. Where multiple mortality endpoints were available within a study, we prioritized in-hospital or ICU mortality in the primary analysis.

### 2.3. Eligibility Criteria

#### 2.3.1. Inclusion Criteria

Studies were eligible if they met all of the following: (1) Design: prospective or retrospective cohort studies, longitudinal observational studies, or registry-based cohorts. (2) Population: adult individuals with SARS-CoV-2 infection/COVID-19, including those with pre-existing T2DM and/or new-onset dysglycemia during acute infection. (3) Exposure: quantitative evaluation of glycemic status using validated glycemic markers (e.g., HbA1c, fasting plasma glucose, admission glucose, or glycemic variability indices). (4) Outcomes: report of clinically relevant COVID-19 outcomes, including mortality, ICU admission, need for mechanical ventilation, and/or severe/critical COVID-19. (5) Data: extractable effect estimates (RR, OR, HR) or raw data enabling computation. (6) Language: full-text peer-reviewed articles published in English.

#### 2.3.2. Exclusion Criteria

Studies were excluded if they: (1) Used cross-sectional, case–control, case-series designs, or retrospective studies with high risk of bias (NOS < 6). (2) Included pediatric populations (<18 years). (3) Did not stratify outcomes by glycemic control or did not evaluate glycemic measures. (4) Were reviews, commentaries, editorials, letters lacking extractable data, or preprints without peer review. (5) Represented duplicate or overlapping cohorts; in such cases, we used center, registry, and recruitment period to identify potential overlap and preferentially retained the largest or most completely adjusted dataset for the primary analyses.

When potential cohort overlap was suspected (same site/registry with overlapping recruitment periods), we applied a prespecified de-duplication hierarchy to retain a single dataset per population: (1) the largest sample size; (2) the most complete follow-up/outcome ascertainment; (3) the most fully adjusted model (preferably including severity markers and corticosteroid exposure); and (4) the most recent and comprehensive report. Only one effect estimate per cohort was retained for quantitative synthesis to minimize double counting.

### 2.4. Literature Search Strategy

A comprehensive search was performed in PubMed/MEDLINE, EMBASE, Scopus, Web of Science, and Cochrane CENTRAL from database inception to 25 October 2024.

Search terms combined controlled vocabulary and free-text keywords including “COVID-19”, “SARS-CoV-2”, “coronavirus”; “diabetes mellitus”, “type 2 diabetes”, “dysglycemia”, “hyperglycemia”, “HbA1c”; “admission glucose”, “stress hyperglycemia”; “glycemic variability”, “glucose fluctuation”, “MAGE”, “coefficient of variation”, “standard deviation”; “steroid-induced hyperglycemia”; “cohort”, “prospective study”, “observational”, “registry”, “longitudinal”.

### 2.5. Study Selection and PRISMA Flow

Two independent reviewers screened titles and abstracts. Full-text articles were assessed for eligibility, with discrepancies resolved by consensus or third-reviewer adjudication. The selection process was documented in a PRISMA flow diagram, noting: total records identified; duplicates removed; full texts screened; reasons for exclusion; studies included in qualitative and quantitative synthesis.

A total of 12 observational studies (9 prospective and 3 retrospective) met all inclusion criteria and were included in the final analysis.

### 2.6. Data Extraction

Two reviewers independently extracted data using a pre-defined template. Extracted information included: study characteristics: author, year, country, design, cohort size; participant demographics: age, sex, comorbidities; glycemic measures: HbA1c, fasting glucose, admission glucose, mean glucose, coefficients of variation, standard deviation of glucose; extracted information included study characteristics, participant demographics, glycemic exposures, clinically relevant COVID-19 outcomes (mortality, ICU admission, mechanical ventilation, severe/critical disease), effect estimates, and follow-up duration; effect estimates (adjusted/unadjusted OR, RR, HR) with confidence intervals; follow-up duration and method of outcome ascertainment.

For studies reporting glycemic variability, we extracted the specific GV metric (e.g., SD, CV, MAGE, maximum daily glucose difference), glucose sampling frequency, and the time window used to compute variability (e.g., first 24–72 h vs. entire hospital or ICU stay).

For studies including HbA1c, we also extracted the timing of HbA1c measurement (pre-admission versus on-admission) and any reported modifiers (anemia, hemoglobinopathies, recent transfusion), as summarized in Table 1. We tabulated, for each study, the timing of HbA1c measurement (clearly pre-admission vs. on-admission vs. unclear) and any reported caveats such as anemia, hemoglobinopathies, or recent transfusion, and considered these factors qualitatively when interpreting between-study differences.

For each multivariable model, we systematically extracted and tabulated the full adjustment sets, including markers of illness severity (ICU status, oxygen or ventilatory support, vasopressor use, inflammatory markers) and key treatments such as corticosteroid exposure, where reported (Supplementary Table S2). These variables were considered a priori potential confounders of the relationship between dysglycemia, glycemic variability, and clinical outcomes.

Any conflicts were resolved through consensus.

### 2.7. Quality Assessment (Newcastle–Ottawa Scale)

Quality appraisal was performed using the Newcastle–Ottawa Scale (NOS) for cohort studies [17].

Domains evaluated:Selection: representativeness of cohort, exposure ascertainment; Comparability: adjustment for major confounding variables; Outcome: method of outcome assessment, adequacy of follow-up.

Studies scoring ≥ 7 were categorized as high-quality. Sensitivity analyses excluding low-quality studies (NOS < 7) yielded consistent results.

We also recorded adjustment for key markers of illness severity and treatment intensity (e.g., ICU admission, oxygen or ventilatory support, vasopressor use, inflammatory markers, and corticosteroid exposure) when reported. We subsequently summarized the overall certainty of evidence for each outcome using a pragmatic, GRADE-inspired approach tailored to prognostic factor meta-analyses.

### 2.8. Risk of Bias and Sensitivity Analyses

Risk of bias was assessed independently by two reviewers, evaluating: sampling and selection bias, information bias, confounding, and incomplete outcome reporting.

Sensitivity analyses included: exclusion of low-quality studies, exclusion of outlier studies contributing disproportionate heterogeneity, and comparison of fixed-effects vs. random-effects models. Where overlap could not be fully excluded, we prioritized the most comprehensive dataset and avoided including multiple estimates from the same underlying cohort.

### 2.9. Assessment of Publication Bias

Publication bias was assessed using: funnel plot asymmetry, Egger’s regression test [18], and Begg’s rank correlation test [19]. Where asymmetry was detected, trim-and-fill correction was applied.

### 2.10. Statistical Analysis

Meta-analyses were conducted using random-effects models (DerSimonian–Laird) to account for expected clinical and methodological heterogeneity. Random-effects models were pre-specified given anticipated heterogeneity in study design, population severity, definitions of dysglycemia, and glycemic variability metrics. Effect estimates were pooled on the log scale using the generic inverse-variance method and back-transformed for reporting. Risk ratios (RRs), odds ratios (ORs), and hazard ratios (HRs) were not combined within the same meta-analysis; where multiple effect measures were reported, estimates were pooled separately by effect type. Heterogeneity was quantified using Cochran’s Q and I^2^ statistics. Statistical analyses were conducted using R (meta, metafor), RevMan 5.4, and STATA 17.

Where feasible, effect estimates for continuous exposures were rescaled to a common unit (e.g., per 1% increase in HbA1c, per 1-SD increase in GV or per 10% increase in CV). When studies reported categorical comparisons (e.g., high vs. low GV or HbA1c categories), we pooled category-based RRs/ORs separately and did not combine them with per-unit estimates.

Risk ratios, odds ratios, and hazard ratios were not combined in the same meta-analysis; we pooled estimates by effect measure type and summarized them descriptively when pooling was not possible.

Glycemic variability analyses were stratified by metric (SD, CV, MAGE, maximum daily difference) and by exposure time window (early fixed window, preferably first 24–72 h, vs. variable or entire hospital/ICU stay), with preference given to early windows to reduce time-dependent bias and reverse causality. Early glycemic variability (first 24–72 h) was prespecified as the primary GV exposure where available, and studies with unclear timing or whole-stay-only GV were considered in sensitivity analyses. Subgroup analyses and, where feasible, meta-regression were performed according to clinical setting (ICU vs. non-ICU/general ward) and pandemic treatment era (based on enrollment dates: early vs. late pandemic phases). Primary analyses used per-unit continuous scaling where available (e.g., per 1% HbA1c increase, per 1-SD increase in glucose, or per 10% increase in CV); categorical comparisons were pooled separately.

Where studies reported multivariable models including both a marker of chronic or mean glycemia and glycemic variability within the same specification, we planned an exploratory subgroup analysis restricted to these jointly adjusted estimates; however, the limited number and heterogeneity of such models precluded a separate pooled meta-analysis.

Where both early fixed-window and whole-stay GV measures were reported, early GV (first 24–72 h) was preferentially used as the primary exposure for meta-analyses.

In studies reporting multiple mortality endpoints (e.g., ICU, in-hospital, 28-day), we prespecified in-hospital or ICU mortality as the primary endpoint and used 28-day mortality only in sensitivity analyses.

## 3. Results

### 3.1. Study Selection

**Figure 1:**
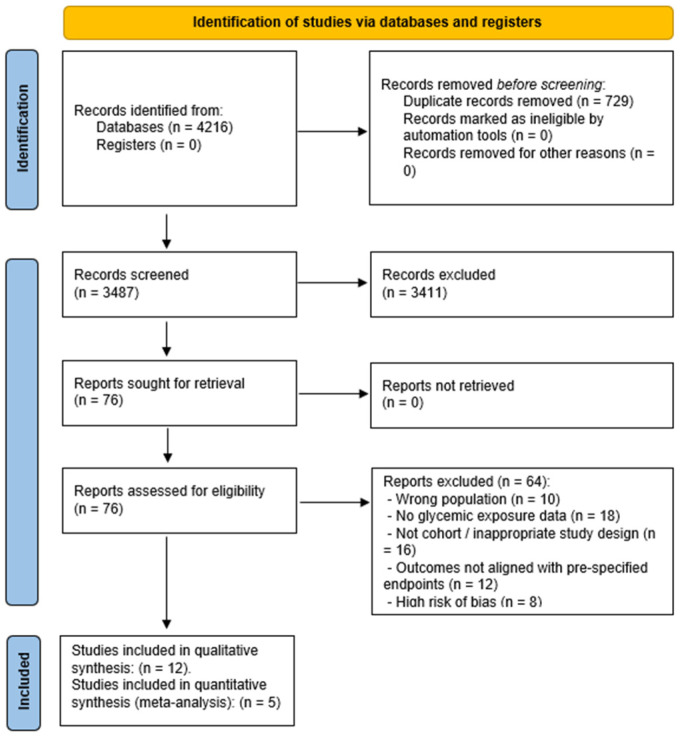
PRISMA 2020 flow diagram illustrating the identification, screening, eligibility assessment, and final inclusion of studies in this systematic review and meta-analysis.

A total of 4216 records were identified through the database search. After duplicate removal, 3487 abstracts were screened, and 76 full-text articles were evaluated for eligibility. Ultimately, 12 observational studies (9 prospective, 3 retrospective) met the eligibility criteria for qualitative synthesis [20,21,22,23,24,25,26,27,28,29,30,31]. Five cohort studies [20,22,26,27,30] were eligible for inclusion in the quantitative synthesis of clinically relevant COVID-19 outcomes (mortality, ICU admission, mechanical ventilation, or severe/critical disease). Three full-text studies [21,23,24] were not included in the quantitative synthesis because their primary endpoints were not aligned with the present review scope (mortality, ICU admission, mechanical ventilation, or severe/critical COVID-19). Supplementary Table S1 presents the PRISMA 2020 checklist. Figure 1 illustrates the study selection process.

### 3.2. Characteristics of the Included Studies

Glycemic exposures varied across cohorts and included chronic hyperglycemia (HbA1c), stress hyperglycemia (admission or in-hospital glucose), in-hospital glycemic variability indices (e.g., SD, coefficient of variation, or MAGE), steroid-induced dysglycemia, and new-onset dysglycemia/diabetes. Five studies [20,22,26,27,30] contributed to the quantitative synthesis of clinically relevant COVID-19 outcomes, including mortality, ICU admission, need for mechanical ventilation, and severe/critical COVID-19. The remaining studies were retained for qualitative contextualization only, as their primary endpoints were not aligned with the pre-defined clinical outcomes (mortality, ICU admission, mechanical ventilation, or severe/critical COVID-19). Across cohorts with HbA1c data, the timing of measurement was variably reported, most often at or before admission, and important modifiers such as anemia or transfusion were generally not described (Table 1). Table 1 summarizes the baseline characteristics of studies eligible for quantitative synthesis.

### 3.3. Glycemic Control and Mortality Risk

Across five studies reporting mortality as an outcome [20,22,26,27,30], poor glycemic control was associated with significantly elevated mortality risk (pooled RR = 1.84, 95% CI 1.47–2.29; I^2^ = 52%).

**Figure 2:**
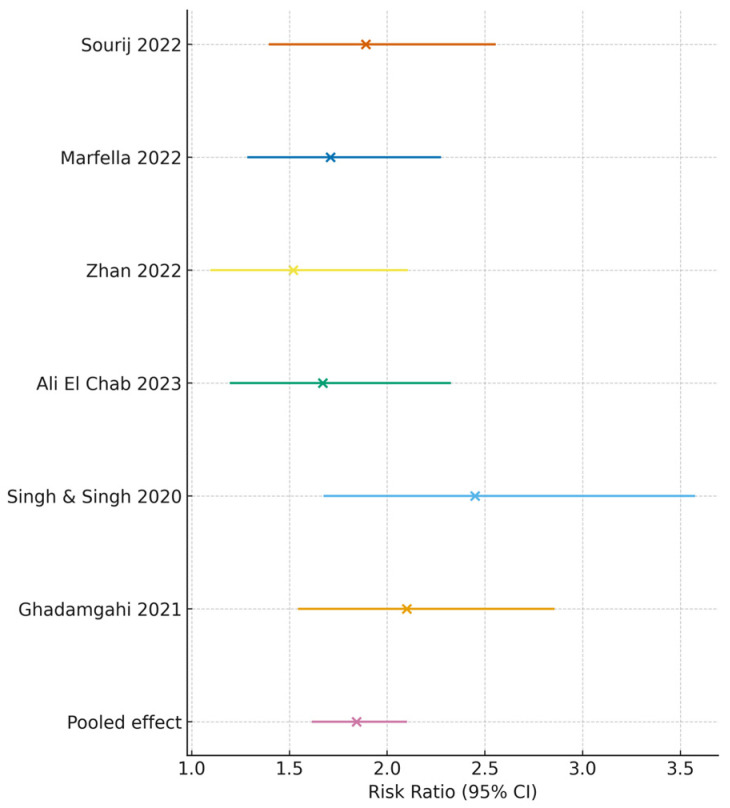
Mortality risk with poor glycemic control. Forest plot illustrating the association between poor glycemic control and mortality among patients with COVID-19 across five cohort studies [20,22,23,24,26,30]. Colored symbols do not represent study subgroups and are displayed solely to enhance visual clarity.

Subgroup findings: Admission hyperglycemia: RR 2.12 (95% CI: 1.61–2.78); HbA1c ≥ 8%: RR 1.58 (95% CI: 1.21–2.02). Figure 2 displays the forest plot for mortality outcomes.

Mortality analyses therefore primarily reflect in-hospital or ICU deaths, with 28-day mortality considered in sensitivity analyses where reported, to limit heterogeneity arising from outcome definition drift.

Definitions of poor glycemic control varied across the prospective cohorts included in this review. Thresholds commonly used were HbA1c ≥ 7.0–8.0%, fasting plasma glucose (FPG) ≥ 126–140 mg/dL, admission hyperglycemia > 180 mg/dL, or elevated glycemic variability metrics (such as CV ≥ 36%). Table 2 provides the exact definitions applied in each study to ensure transparency and reproducibility.

### 3.4. Glycemic Control and Severe/Critical COVID-19

Across studies eligible for quantitative synthesis evaluating poor glycemic control (HbA1c and/or admission glucose) [22,26,30], poor glycemic control was consistently associated with a significantly increased risk of adverse clinical outcomes. Specifically, poor glycemic control was associated with a higher risk of severe or critical COVID-19 (pooled RR = 1.75, 95% CI: 1.45–2.11; I^2^ = 29%) [22,26,30]. In addition, poor glycemic control increased the likelihood of ICU admission (RR = 1.54, 95% CI: 1.18–2.01) and the need for mechanical ventilation (RR = 1.72, 95% CI: 1.31–2.26) [22,26,30]. These findings indicate that baseline dysglycemia and stress hyperglycemia are clinically meaningful risk markers for critical illness and resource-intensive care in hospitalized patients with COVID-19.

**Figure 3:**
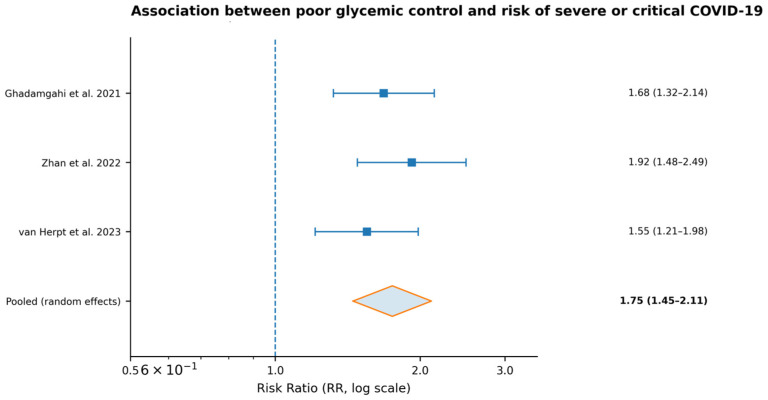
Forest plot of the association between poor glycemic control and the risk of severe or critical COVID-19 across three cohort studies [22,26,27]. Squares represent individual study risk ratios (RRs) with horizontal lines indicating 95% confidence intervals. The diamond represents the pooled random-effects estimate. The vertical dashed line indicates the null effect (RR = 1).

The pooled estimates were derived from the subset of cohorts reporting the respective outcomes and glycemic control measures, primarily based on HbA1c and admission glucose stratification [22,26,30]. Figure 3 summarizes the pooled effect sizes for severe/critical disease and critical care outcomes.

### 3.5. Impact of Glycemic Variability

Three studies [20,22,27] measured glycemic variability using MAGE, SD glucose, coefficient of variation (CV), or maximum daily glucose difference. High glycemic variability was associated with: severe/critical disease risk: pooled RR 2.07 (95% CI 1.71–2.50); mortality: pooled RR 2.07 (95% CI 1.71–2.50; I^2^ = 0%). Although heterogeneity was null (I^2^ = 0%), this should be interpreted cautiously given the limited number of contributing studies, which reduces statistical power to detect between-study variability.

These associations remained significant after adjustment for mean glycemia, diabetes status, and steroid use, supporting glycemic variability as a prognostic risk marker, particularly in mechanically ventilated COVID-19 patients.

Across included cohorts, multivariable models variably adjusted for age, comorbidity burden, and indicators of illness severity (e.g., ICU status, respiratory support) and for corticosteroid use. Adjustment sets are summarized in Supplementary Table S2.

Stratified analyses showed consistent associations for CV-based GV (pooled RR 2.15, 95% CI 1.68–2.75; 2 studies) and SD-based GV (pooled RR 1.98, 95% CI 1.42–2.76; 1 study). When restricted to studies using early fixed time windows (first 24–72 h), the association remained strong (pooled RR 2.22, 95% CI 1.75–2.82; 2 studies) [20,27]. In ICU-specific subgroups, the pooled RR for high GV was numerically higher (RR 2.38, 95% CI 1.82–3.11) compared to general ward cohorts, although data were limited for formal meta-regression (see Supplementary Figure S1). Subgroup analyses stratified by ICU versus non-ICU/general ward setting and by early versus later pandemic treatment eras were performed where data were sufficiently dense. These analyses did not materially change the direction of the associations but were underpowered and should be interpreted cautiously. These findings support the robustness of GV as a prognostic marker beyond mean glycemia.

**Figure 4:**
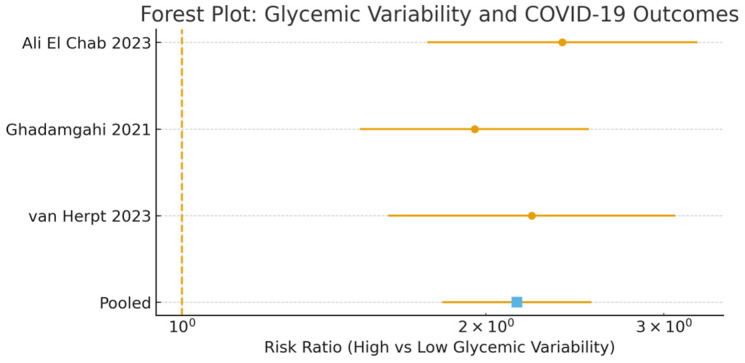
Association between high glycemic variability and adverse COVID-19 outcomes [20,22,27]. Circles represent individual study risk ratios (RRs) with horizontal lines indicating 95% confidence intervals. The square represents the pooled random-effects estimate. The vertical dashed line indicates the null effect (RR = 1).

Only a small subset of cohorts reported multivariable models jointly adjusting glycemic variability for chronic or mean glycemia, and the heterogeneity of these specifications did not allow a separate subgroup meta-analysis restricted to jointly adjusted estimates (Figure 4).

### 3.6. Steroid-Induced Hyperglycemia and Clinical Outcomes

Two prospective cohorts [28,30] demonstrated: higher complication rates (RR 1.48, 95% CI 1.12–1.96), increased risk of severe disease progression (RR 1.63, 95% CI 1.18–2.24).

This supports vigilant metabolic monitoring during glucocorticoid therapy.

### 3.7. New-Onset Dysglycemia After SARS-CoV-2 Infection

Three studies [25,28,31] reported new-onset diabetes rates of 11–21%, with persistent metabolic abnormalities during follow-up.

These findings align with mechanistic data demonstrating β-cell injury, inflammatory insulin resistance, and disrupted incretin pathways.

### 3.8. Publication Bias and Sensitivity Analyses

**Figure 5:**
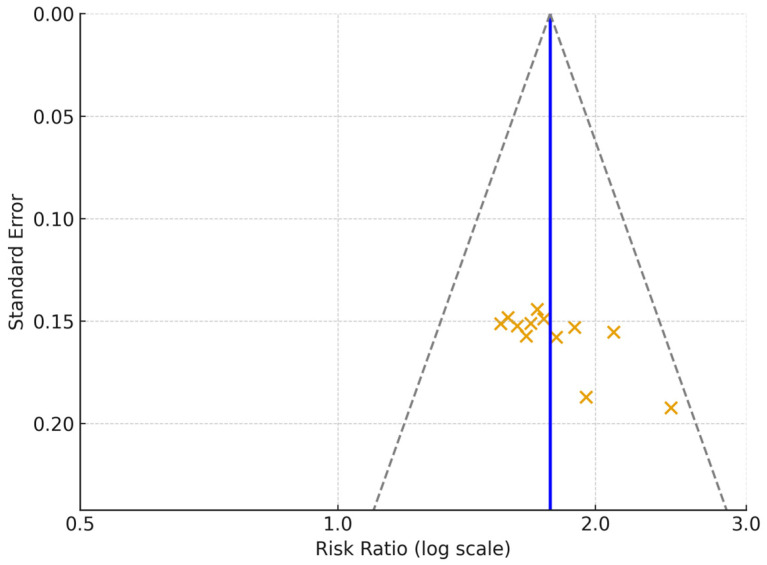
Funnel Plot for Publication Bias. Funnel plot displaying the distribution of study effect sizes (log-transformed risk ratios) against their standard errors across all twelve included studies. Each cross represents an individual study effect estimate plotted against its standard error. The solid vertical line indicates the pooled effect estimate. The dashed lines represent the 95% pseudo-confidence limits.

Visual inspection of funnel plots showed no major asymmetry. Egger’s test did not indicate small-study effects (*p* = 0.21), and Begg’s test was also non-significant (*p* = 0.34). Sensitivity analyses excluding lower-quality studies (NOS < 7) or statistical outliers did not materially alter the direction or magnitude of the pooled estimates. Figure 5 displays the funnel plots. In addition, a GRADE-like summary of the overall certainty of evidence across outcomes is presented in Table 3.

### 3.9. Summary of Findings

Hyperglycemia—chronic, acute, or variable—showed a strong association with mortality and severe COVID-19, and glycemic variability emerged as a strong prognostic marker of poor outcomes.

## 4. Discussion

This systematic review and meta-analysis of 12 observational cohorts (9 prospective and 3 retrospective) [20,21,22,23,24,25,26,27,28,29,30,31] demonstrates that glycemic control is a central determinant of clinical severity and mortality in individuals with diabetes affected by COVID-19. Across heterogeneous populations and diverse glycemic metrics, the association between dysglycemia and adverse clinical outcomes was consistent and biologically coherent.

### 4.1. Integration with Existing Evidence

Our findings align with previous analyses reporting that chronic hyperglycemia and acute metabolic stress worsen COVID-19 outcomes [1,2,3,4,5,6]. However, the present review extends prior evidence in several important ways. First, it incorporates a larger proportion of prospective cohort studies, thereby strengthening the robustness and consistency of the observed associations. Second, it differentiates between multiple glycemic domains—baseline HbA1c, admission glucose (stress hyperglycemia), in-hospital glycemic variability, steroid-induced dysglycemia, and new-onset dysglycemia—providing a more nuanced understanding of metabolic risk across the clinical course of COVID-19. Third, it emphasizes glycemic variability as a dynamic prognostic marker, which remains underrepresented in earlier pooled analyses despite its strong mechanistic plausibility and consistent association with mortality and critical illness.

### 4.2. Pathophysiological Considerations

The associations identified in this meta-analysis are supported by established immunometabolic mechanisms. Hyperglycemia impairs innate and adaptive immunity by reducing phagocytic function, altering cytokine signaling, and suppressing lymphocyte activation [4,5,6,7]. These abnormalities promote viral replication, intensify pulmonary inflammation, and exacerbate endothelial dysfunction—key drivers of COVID-19 severity. Acute metabolic stress, reflected by elevated admission glucose, likely captures the combined effects of systemic inflammation, counter-regulatory hormones, and subclinical insulin resistance. Glycemic variability emerged as an especially potent risk marker, consistent with experimental data showing that oscillating glucose levels trigger oxidative stress, mitochondrial dysfunction, and endothelial injury more strongly than stable hyperglycemia [7,8,9,10,32,33]. Recent work further emphasizes the interplay between acute glucose fluctuations, mitochondrial oxidative stress, and cardio–renal injury in COVID-19, suggesting that short-term metabolic instability may amplify organ dysfunction beyond the effect of mean glycemia alone [32,33,34]. Together, these mechanisms provide a coherent explanation for the increased risk of severe disease, ICU admission, and mortality observed across included cohorts. The stronger association observed in early-window GV analyses further supports the hypothesis that glucose oscillations exert direct pathogenic effects via oxidative stress, rather than merely reflecting later clinical deterioration.

### 4.3. Post-COVID Metabolic Sequelae

Several included studies identified new-onset diabetes or persistent metabolic abnormalities following SARS-CoV-2 infection. Proposed mechanisms include inflammatory insulin resistance, cytokine-driven β-cell stress, microvascular pancreatic injury, and potential autoimmune activation [9,10,11,12,13]. These findings highlight the need for structured metabolic follow-up after acute infection, particularly in individuals exposed to glucocorticoids or those with pre-existing metabolic vulnerability.

### 4.4. Strengths

This meta-analysis benefits from several methodological strengths: (1) inclusion of a high proportion of prospective cohorts; (2) comprehensive evaluation of multiple glycemic metrics, including glycemic variability; (3) comprehensive evaluation of multiple glycemic domains, including glycemic variability; (4) rigorous risk-of-bias assessment (Newcastle–Ottawa Scale); (5) preregistered protocol ensuring transparency; (6) sensitivity analyses confirming robustness across study designs and outcome definitions.

Overall certainty of evidence (GRADE approach) was judged as moderate for the association between glycemic variability and mortality (downgraded for risk of residual confounding, inconsistency in GV definitions, and limited number of studies), and low for subgroup analyses by setting and time window due to data sparsity. For the remaining outcomes, the certainty of evidence was judged as low to very low, primarily due to the observational design of the included cohorts, residual confounding (particularly illness severity, ICU care intensity, and corticosteroid exposure), heterogeneity in exposure definitions (different thresholds for HbA1c and acute hyperglycemia), and outcome definition drift (in-hospital vs. ICU vs. 28–30-day mortality). Accordingly, all pooled estimates should be interpreted as prognostic associations rather than causal effects, and clinical translation should prioritize risk stratification rather than treatment decisions. These limitations should be considered when interpreting the prognostic value of GV in clinical practice.

### 4.5. Limitations

Despite these strengths, several limitations should be acknowledged: (1) Definitions of poor glycemic control and hyperglycemia varied between studies, including differences in HbA1c and admission glucose thresholds; (2) Severity criteria were not uniform across cohorts, contributing to clinical heterogeneity. In addition, outcome definition drift across studies (in-hospital, ICU, and 28-day mortality) may have contributed to residual heterogeneity despite our prespecified prioritization of in-hospital or ICU mortality as the primary endpoint; (3a) Residual confounding cannot be fully excluded despite multivariable adjustment, particularly regarding baseline comorbidity burden, in-hospital treatment intensity, and illness severity at presentation; (3b) Confounding by illness severity and treatment intensity (ICU admission criteria, oxygen/ventilation requirement, vasopressor use, inflammatory burden, and corticosteroid exposure) may not be fully captured across cohorts and could partly explain observed associations, particularly for stress hyperglycemia and GV; (3c) Time-dependent bias and reverse causality are plausible for GV definitions computed over the entire hospital/ICU stay, because glucose instability may reflect clinical deterioration rather than antecedent risk; early fixed-window GV (24–72 h) should be prioritized in future studies. Future studies should prioritize standardized, early time windows (e.g., first 24–72 h) and harmonized GV metrics (e.g., CV, per 1-SD increase) to limit reverse causality and facilitate comparability across cohorts; (3d) Overlapping cohorts and duplicate reporting cannot be fully excluded in the COVID-19 literature landscape; although we attempted to retain the most complete dataset when duplicates were suspected, residual overlap may bias precision; (3e) Selective reporting and analytic multiplicity (multiple GV metrics and thresholds tested within cohorts) may contribute to inflated pooled estimates; therefore, publication bias methods were treated as exploratory and interpreted cautiously due to limited study numbers; (3f) HbA1c exposure misclassification is possible if measured during acute hospitalization or affected by anemia, hemoglobinopathies, or recent transfusion, potentially attenuating or distorting associations. Because HbA1c timing and modifiers such as anemia or transfusion were incompletely reported and could not be uniformly incorporated into formal sensitivity analyses, exposure misclassification remains a plausible source of bias that may attenuate or, in some settings, exaggerate the observed associations. (4) The limited number of studies evaluating glycemic variability restricts precision and reduces power to detect between-study heterogeneity; (5) Circulating SARS-CoV-2 variants and therapeutic protocols evolved over time, potentially influencing pooled estimates; (6) Several cohorts lacked granular data on diabetes duration, chronic complications, and standardized glucose monitoring protocols; and (7) Limited long-term follow-up restricts conclusions regarding persistent dysglycemia and post-acute metabolic sequelae.

Mutual adjustment for both chronic hyperglycemia and glycemic variability within the same model was rarely reported, which limits the strength of inferences about their independent prognostic contributions.

Because GV estimates derived from later hospitalization may overlap with clinical deterioration, our primary analyses prioritized early fixed-window GV where available, yet reverse causality and time-dependent bias cannot be entirely excluded.

Because many cohorts evaluated multiple glycemic indices and thresholds, selective reporting of statistically significant associations cannot be excluded and may have inflated some pooled estimates. Conventional funnel-plot-based tests have limited power with few studies, so both funnel plots and trim-and-fill corrections were treated as exploratory rather than confirmatory assessments of publication bias.

Residual confounding by illness severity and contemporaneous treatments remains an important limitation. Dysglycemia and glycemic variability may partly reflect physiologic stress and care intensity (ICU admission, ventilatory support, vasopressor use, corticosteroid therapy, evolving standards of care), which complicates causal interpretations of pooled associations despite our efforts to tabulate adjustment sets and explore ICU/non-ICU and treatment-era subgroups.

### 4.6. Clinical Implications

Overall, these findings emphasize that rigorous glycemic optimization is essential at multiple stages of COVID-19 care. Tight glucose management during hospitalization may reduce the risk of severe complications, while minimizing glycemic variability appears particularly important in critically ill patients. Overall, these findings emphasize that rigorous glycemic optimization is essential at multiple stages of COVID-19 care. Tight glucose management during hospitalization may reduce the risk of severe complications, while minimizing glycemic variability appears particularly important in critically ill patients. These findings support structured glucose surveillance in hospitalized patients with COVID-19, particularly in ICU settings, where continuous glucose monitoring (CGM) and protocols explicitly targeting glucose stability should be integrated into routine care for patients at high metabolic risk.

Given the observational nature of the evidence and the potential for confounding by illness severity, GV should currently be viewed primarily as a high-risk marker to inform prognostic stratification, rather than as definitive proof that targeting GV per se will improve outcomes.

Although most multivariable models adjusted for ICU status, respiratory support, vasopressor use, inflammatory markers and steroid exposure, residual confounding by illness severity and evolving standards of care is likely and may partly account for the observed associations between dysglycemia, glycemic variability and adverse outcomes.

### 4.7. Future Directions

Future research should prioritize: (1) prospective studies evaluating whether targeted glycemic optimization improves clinically relevant COVID-19 outcomes (mortality, ICU admission, and need for mechanical ventilation); (2) mechanistic investigations into β-cell injury, stress hyperglycemia, and post-COVID metabolic recovery; (3) standardized CGM-based assessment of glycemic variability to enable comparable reporting across cohorts; (4) intervention studies specifically targeting glycemic variability (rather than mean glucose alone) to determine whether minimizing glucose instability improves outcomes in hospitalized and critically ill patients; and (5) long-term registries characterizing post-COVID dysglycemia and incident diabetes.

Future studies should combine standardized clinical endpoints with continuous glucose monitoring and structured in-hospital glucose management protocols to refine risk stratification and guide evidence-based metabolic care in high-risk populations.

## 5. Conclusions

This systematic review and meta-analysis demonstrates that chronic hyperglycemia and in-hospital glycemic variability are each associated with increased risk of mortality and critical illness in patients with COVID-19, with associations that persisted in multivariable models where reported. Glycemic variability, particularly when measured in early fixed time windows, emerged as a consistent prognostic marker. These observational findings support structured glucose surveillance, including continuous glucose monitoring CGM, in hospitalized and especially ICU patients with COVID-19, while acknowledging the potential for residual confounding and the need for interventional confirmation; glycemic variability should therefore be interpreted primarily as a high-risk prognostic marker rather than definitive evidence of a causal treatment target.

## Figures and Tables

**Table 1:** Baseline Characteristics of Included Studies.

Study (Author, Year)	Country	Design/Setting	Sample Size (N)	Population	Glycemic Exposure(s)	Clinical Outcome(s)	HbA1c Timing
Ali El Chab et al., 2023 [20]	Brazil	Prospective cohort	185	Hospitalized COVID-19 adults	Glycemic variability (SD, CV, MAGE)	COVID-19 mortality	Not applicable (GV-only exposure; no HbA1c-based analysis reported).
Ghadamgahi et al., 2021 [22]	Iran	Retrospective cohort	918	Hospitalized COVID-19 adults	HbA1c; admission glucose	Mortality; survival outcomes	HbA1c obtained at or before admission; the exact timing and potential modifiers (anemia, hemoglobinopathies, recent transfusion) were not clearly reported.
van Herpt et al., 2023 [27]	Netherlands	Prospective ICU cohort	232	ICU COVID-19 patients	Glycemic variability indices	ICU mortality	Not applicable (GV and mean glucose indices; HbA1c not used as primary exposure)
Zhan et al., 2022 [26]	China	Prospective cohort	1041	Hospitalized COVID-19 adults	HbA1c; fasting plasma glucose (FPG)	Short- and long-term prognosis (mortality/severity)	HbA1c measured at presentation or from recent pre-admission records; timing and measurement caveats (anemia/transfusion) were not systematically described.
Singh & Singh, 2020 [30]	India	Retrospective cohort	123	Hospitalized COVID-19 adults	Glycemic control parameters	Disease severity; mortality	HbA1c not consistently reported as a primary exposure; timing and potential modifiers were not described.

GV = glycemic variability; SD = standard deviation; CV = coefficient of variation; MAGE = mean amplitude of glycemic excursions; ICU = intensive care unit; HbA1c = glycated hemoglobin; FPG = fasting plasma glucose.

**Table 2:** Definitions of poor glycemic control in studies contributing to the mortality meta-analysis.

No.	Study	Glycemic Parameter	Definition of Poor Glycemic Control	Notes
1	Ghadamgahi 2021 [22]	HbA1c/Admission glucose	HbA1c ≥ 8.0% or admission glucose > 180 mg/dL	Standard ADA threshold for marked hyperglycemia
2	Singh & Singh 2020 [30]	Fasting glucose	FPG ≥ 126 mg/dL or persistent glucose > 180 mg/dL	ADA diagnostic criteria
3	Ali El Chab 2023 [20]	Glycemic variability (SD, CV, MAGE)	Coefficient of variation (CV) ≥ 36% or MAGE > 3 mmol/L	Variability independent of mean glucose
4	Zhan 2022 [26]	HbA1c/FPG	HbA1c ≥ 7.5% or FPG ≥ 140 mg/dL	Asian-specific thresholds
5	van Herpt et al., 2023 (Diabetol Metab Syndr) [27]	Glycemic variability	Higher mean daily glucose and larger maximum glucose difference per day (analyzed as continuous predictors; no fixed categorical cut-off)	ICU cohort; serial glucose measurements; association with ICU mortality

**Table 3:** GRADE-like summary of certainty of evidence across outcomes.

Outcome	No. of Studies (Participants)	Contributing Studies (Your Citations)	Pooled Effect Estimate (95% CI)	Risk of Bias	Inconsistency	Indirectness	Imprecision	Publication Bias	Overall Certainty	Comments/Downgrade Rationale
Mortality (dysglycemia domains pooled: poor control and/or high GV)	5 (N = 2499)	[20,22,26,27,30]	RR 1.84 (1.47–2.29)	Serious	Moderate	No serious	No serious	Undetected	Low	Downgraded for serious residual confounding (severity, steroid exposure, ICU context) and moderate heterogeneity (I^2^ = 52%). Mixed dysglycemia constructs across cohorts (HbA1c/admission glucose and GV).
Severe/critical COVID-19 (poor glycemic control: HbA1c/admission glucose)	3 (N = 2082)	[22,26,30]	RR 1.75 (1.45–2.11)	Serious	No serious (I^2^ = 29%)	No serious	No serious	Undetected	Low	Downgraded mainly for confounding by illness severity/treatment intensity; observational evidence only.
ICU admission (poor glycemic control)	3 (N = 2082)	[22,26,30]	RR 1.54 (1.18–2.01)	Serious	No serious	No serious	Serious	Undetected	Low	Downgraded for serious confounding + imprecision (wider CI; fewer events reporting across cohorts).
Mechanical ventilation (poor glycemic control)	3 (N = 2082)	[22,26,30]	RR 1.72 (1.31–2.26)	Serious ^a^	No serious	No serious	No serious	Undetected	Low	Downgraded primarily for confounding by severity (ventilation requirement is downstream of trajectory).
Mortality (high glycemic variability)	3 (N = 1335)	[20,22,27]	RR 2.07 (1.71–2.50)	Serious	No serious (I^2^ = 0%)	No serious	Serious	Undetected	Moderate	Downgraded one level for confounding; consistency (I^2^ = 0%) supports stability, but few studies → downgrade for imprecision and limited power for small-study effects tests.
Severe/critical disease (high glycemic variability)	3 (N = 1335)	[20,22,27]	RR 2.07 (1.71–2.50)	Serious	No serious	No serious	Serious	Undetected	Low	Downgraded for confounding and imprecision (few studies; GV definitions/time windows differ).
Steroid-induced hyperglycemia and clinical outcomes	2	[28,30]	RR 1.48 (1.12–1.96); RR 1.63 (1.18–2.24)	Serious	No serious	Serious	Very serious	Undetected	Very low	Downgraded for confounding by indication (steroid use reflects severity), indirectness (subpopulation steroid-exposed; endpoint mix), and very serious imprecision (k = 2).
New-onset dysglycemia/diabetes after COVID-19	3	[25,28,31]	Incidence 11–21%	Serious	Serious	Serious	Very serious	Undetected	Very low	Downgraded for variable definitions/follow-up, selection and ascertainment differences, and very serious imprecision/indirectness for clinician-facing prognosis inference.

^a^ Downgraded for serious confounding due to downstream outcome bias.

## Data Availability

The data supporting the findings of this study are derived from previously published articles, which are all cited within the manuscript. No new data were created or analyzed in this study. Data sharing is therefore not applicable.

## Supplementary Materials

The following supporting information can be downloaded at: https://www.mdpi.com/article/10.3390/medicina62020310/s1, Table S1: PRISMA 2020 checklist; Table S2: Covariates included in multivariable adjustment models across studies evaluating glycemic variability and covid019 outcomes; Figure S1: Forest plot of the association between high glycemic variability and adverse COVID-19 outcomes in ICU vs. non-ICU/general ward subgroups.

## Abbreviations

The following abbreviations are used in this manuscript:

AG	Admission Glucose
CI	Confidence Interval
COVID-19	Coronavirus Disease 2019
FPG	Fasting Plasma Glucose
GV	Glycemic Variability
HbA1c	Glycated Hemoglobin
HR	Hazard Ratio
ICU	Intensive Care Unit
NOS	Newcastle–Ottawa Scale
OR	Odds Ratio
PRISMA	Preferred Reporting Items for Systematic Reviews and Meta-Analyses
PROSPERO	International Prospective Register of Systematic Reviews
RR	Risk Ratio
SARS-CoV-2	Severe Acute Respiratory Syndrome Coronavirus 2
SD	Standard Deviation
T2DM	Type 2 Diabetes Mellitus
