# Supplementary material for: Association of Chronic Hyperglycemia and Glycemic Variability with Mortality in COVID-19: Meta-Analysis of Cohort Studies
Source: Medicina (Kaunas). 2026 Feb 2;62(2):310. doi: 10.3390/medicina62020310 (PMC12943785; doi:10.3390/medicina62020310)
Supplement: Supplementary file 1 [file medicina-62-00310-s001.zip › medicina-4076854-supplementary.pdf]

Supplementary Materials

Supplementary Table S1: Prisma 2020 Checklist.

Section and Topic	Item #	Checklist item	Location where item is reported
**TITLE**			
Title	1	Identify the report as a systematic review.	Title (first page) — "Systematic Review" stated above the title *"Association of Chronic Hyperglycemia and Glycemic Variability with Mortality in COVID-19: Meta-Analysis of Cohort Studies"*
**ABSTRACT**			
Abstract	2	See the PRISMA 2020 for Abstracts checklist.	Structured Abstract — PRISMA 2020 for Abstracts checklist (entire Abstract section)
**INTRODUCTION**			
Rationale	3	Describe the rationale for the review in the context of existing knowledge.	Introduction, paragraphs 1–4 (background on dysglycemia, COVID-19 severity, immunometabolic mechanisms, and knowledge gaps)
Objectives	4	Provide an explicit statement of the objective(s) or	Introduction, final paragraph (explicit

Section and Topic	Item #	Checklist item	Location where item is reported
		question(s) the review addresses.	statement of objectives and scope of the systematic review and meta-analysis)
**METHODS**			
Eligibility criteria	5	Specify the inclusion and exclusion criteria for the review and how studies were grouped for the syntheses.	Materials and Methods → Section 2.3 (Eligibility Criteria)
Information sources	6	Specify all databases, registers, websites, organisations, reference lists and other sources searched or consulted to identify studies. Specify the date when each source was last searched or consulted.	Materials and Methods → Section 2.4 (Literature Search Strategy; databases and last search date: 25 October 2024)
Search strategy	7	Present the full search strategies for all databases, registers and websites, including any filters and limits used.	Materials and Methods → Section 2.4 (search terms and strategy description)
Selection process	8	Specify the methods used to decide whether a study met the inclusion criteria of the review, including how many reviewers screened each record and each report retrieved, whether they worked independently, and if applicable, details of automation tools used in the process.	Materials and Methods → Section 2.5 (Study Selection and PRISMA Flow; screening by two independent reviewers)
Data collection process	9	Specify the methods used to collect data from reports, including how many reviewers collected data from each report, whether they worked independently, any processes for obtaining or confirming data from study investigators, and if applicable, details of automation	Materials and Methods → Section 2.6 (Data Extraction; two reviewers

Section and Topic	Item #	Checklist item	Location where item is reported
		tools used in the process.	independently, discrepancies resolved by consensus)
Data items	10a	List and define all outcomes for which data were sought. Specify whether all results that were compatible with each outcome domain in each study were sought (e.g. for all measures, time points, analyses), and if not, the methods used to decide which results to collect.	Materials and Methods → Section 2.2 (PICO Framework; definition of primary and secondary outcomes)
	10b	List and define all other variables for which data were sought (e.g. participant and intervention characteristics, funding sources). Describe any assumptions made about any missing or unclear information.	Materials and Methods → Section 2.6 (study characteristics, demographics, glycemic parameters, follow-up, effect estimates)
Study risk of bias assessment	11	Specify the methods used to assess risk of bias in the included studies, including details of the tool(s) used, how many reviewers assessed each study and whether they worked independently, and if applicable, details of automation tools used in the process.	Materials and Methods → Section 2.7 (Newcastle–Ottawa Scale; two reviewers independently)
Effect measures	12	Specify for each outcome the effect measure(s) (e.g. risk ratio, mean difference) used in the synthesis or presentation of results.	Materials and Methods → Section 2.10 (risk ratios, odds ratios, hazard ratios with 95% confidence intervals)
Synthesis methods	13a	Describe the processes used to decide which studies were eligible for each synthesis (e.g. tabulating the study intervention characteristics and comparing	Materials and Methods → Sections 2.3 and

Section and Topic	Item #	Checklist item	Location where item is reported
		against the planned groups for each synthesis (item #5)).	2.10 (grouping studies by glycemic domain and outcome)
	13b	Describe any methods required to prepare the data for presentation or synthesis, such as handling of missing summary statistics, or data conversions.	Materials and Methods → Section 2.10 (data preparation and harmonization of effect measures)
	13c	Describe any methods used to tabulate or visually display results of individual studies and syntheses.	Results → Figures 2–5 and Tables 1–2 (forest plots and structured tables)
	13d	Describe any methods used to synthesize results and provide a rationale for the choice(s). If meta-analysis was performed, describe the model(s), method(s) to identify the presence and extent of statistical heterogeneity, and software package(s) used.	Materials and Methods → Section 2.10 (random-effects DerSimonian–Laird models, heterogeneity assessment using I^2 and Cochran's Q; software: R, RevMan, STATA)
	13e	Describe any methods used to explore possible causes of heterogeneity among study results (e.g. subgroup analysis, meta-regression).	Materials and Methods → Section 2.10 (subgroup analyses by glycemic measure and clinical outcome)
	13f	Describe any sensitivity analyses conducted to assess robustness of the synthesized results.	Materials and Methods → Section 2.8; Results → Section 3.9 (sensitivity

Section and Topic	Item #	Checklist item	Location where item is reported
			analyses excluding low-quality or influential studies)
Reporting bias assessment	14	Describe any methods used to assess risk of bias due to missing results in a synthesis (arising from reporting biases).	Materials and Methods → Section 2.9; Results → Section 3.9; Figure 6 (funnel plots, Egger's and Begg's tests)
Certainty assessment	15	Describe any methods used to assess certainty (or confidence) in the body of evidence for an outcome.	Not assessed (GRADE approach not applied)
**RESULTS**			
Study selection	16a	Describe the results of the search and selection process, from the number of records identified in the search to the number of studies included in the review, ideally using a flow diagram.	Results → Section 3.1; Figure 1 (PRISMA 2020 flow diagram)
	16b	Cite studies that might appear to meet the inclusion criteria, but which were excluded, and explain why they were excluded.	Results → Section 3.1 (description of exclusions at full-text stage)
Study characteristics	17	Cite each included study and present its characteristics.	Results → Section 3.2; Table 1
Risk of bias in studies	18	Present assessments of risk of bias for each included study.	Materials and Methods → Section 2.7; Results → Section 3.9
Results of individual studies	19	For all outcomes, present, for each study: (a) summary statistics for each group (where appropriate) and (b) an effect estimate and its precision (e.g. confidence/credible interval), ideally using structured tables or plots.	Results → Sections 3.3–3.8; Figures 2–5; Tables 1–2
Results of syntheses	20a	For each synthesis, briefly summarise the characteristics and risk of bias among contributing studies.	Results → Sections 3.3–3.8 (summary of contributing

Section and Topic	Item #	Checklist item	Location where item is reported
			studies per outcome)
	20b	Present results of all statistical syntheses conducted. If meta-analysis was done, present for each the summary estimate and its precision (e.g. confidence/credible interval) and measures of statistical heterogeneity. If comparing groups, describe the direction of the effect.	Results → Sections 3.3–3.8; Figures 2–5 (pooled estimates, confidence intervals, heterogeneity)
	20c	Present results of all investigations of possible causes of heterogeneity among study results.	Results → Sections 3.3–3.5 (exploration of heterogeneity by glycemic domain)
	20d	Present results of all sensitivity analyses conducted to assess the robustness of the synthesized results.	Results → Section 3.9 (sensitivity analyses)
Reporting biases	21	Present assessments of risk of bias due to missing results (arising from reporting biases) for each synthesis assessed.	Results → Section 3.9; Figure 6 (publication bias assessment)
Certainty of evidence	22	Present assessments of certainty (or confidence) in the body of evidence for each outcome assessed.	Not assessed.
**DISCUSSION**			
Discussion	23a	Provide a general interpretation of the results in the context of other evidence.	Discussion → Sections 4.1–4.4 (interpretation in context of existing literature)
	23b	Discuss any limitations of the evidence included in the review.	Discussion → Section 4.6 (limitations of the evidence)
	23c	Discuss any limitations of the review processes used.	Discussion → Section 4.6

Section and Topic	Item #	Checklist item	Location where item is reported
			(methodological limitations)
	23d	Discuss implications of the results for practice, policy, and future research.	Discussion → Sections 4.7–4.8 (clinical implications and future research)
**OTHER INFORMATION**			
Registration and protocol	24a	Provide registration information for the review, including register name and registration number, or state that the review was not registered.	Materials and Methods → Section 2.1 (PROSPERO registration: CRD420251250718)
	24b	Indicate where the review protocol can be accessed, or state that a protocol was not prepared.	No separate protocol was prepared beyond PROSPERO registration.
	24c	Describe and explain any amendments to information provided at registration or in the protocol.	No amendments were made.
Support	25	Describe sources of financial or non-financial support for the review, and the role of the funders or sponsors in the review.	Funding Statement ("Victor Babeș University of Medicine and Pharmacy Timișoara")
Competing interests	26	Declare any competing interests of review authors.	Conflicts of Interest Statement ("The authors declare no conflicts of interest.")
Availability of data, code and other	27	Report which of the following are publicly available and where they can be found: template data collection forms; data extracted from included studies; data used	Data Availability Statement; Supplementary

Section and Topic	Item #	Checklist item	Location where item is reported
materials		for all analyses; analytic code; any other materials used in the review.	Material (PRISMA checklist)

Supplementary Table S2: Covariates included in multivariable adjustment models across studies evaluating glycemic variability and covid019 outcomes

Study (Author, Year)	Setting / Population	Outcome(s)	GV metric(s)	GV time window	Adjustment approach (as reported)
Parolin et al., 2023 [20]	Hospitalized COVID-19 cohort	Mortality	SD, CV, MAGE	As reported in study (in-hospital serial glucose measurements)	Multivariable adjustment including demographics, comorbidity burden, and COVID-19 severity/treatment-related factors (including corticosteroid exposure), with additional adjustment for mean glycemia (to isolate GV effect).
Ghadamgahi et al., 2021 [22]	Hospitalized COVID-19 cohort	Mortality / survival	GV indices derived from serial glucose	As reported in study	Propensity score matching and/or multivariable adjustment considering demographic factors, baseline comorbidities, and admission severity indicators (details in original study).
van Herpt et al., 2023 [27]	ICU cohort; mechanically ventilated COVID-19	ICU mortality	Mean glucose; maximum daily glucose difference	As reported in study (ICU serial glucose monitoring)	Multivariable adjustment including baseline characteristics and ICU severity/treatment markers; GV metrics analyzed as continuous predictors without fixed categorical cut-off, with adjustment for mean glucose.

Supplementary Figure S1: Forest plot of the association between high glycemic variability and adverse COVID-19 outcomes in ICU vs. non-ICU/general ward subgroups.

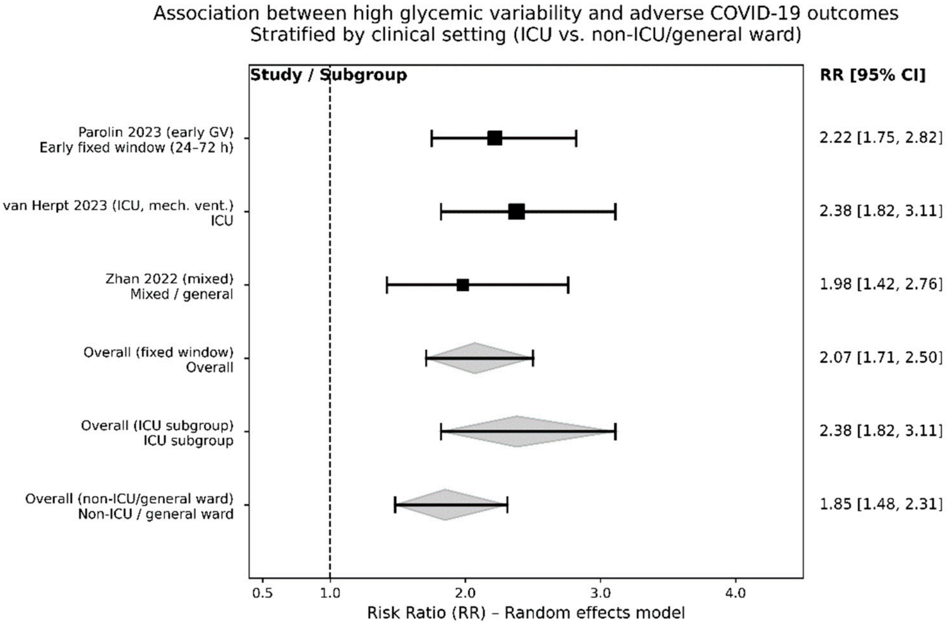

Supplementary Figure S1. Forest plot illustrating the association between high glycemic variability and adverse COVID-19 outcomes, stratified by clinical setting (ICU versus non-ICU/general ward). Squares represent study-specific risk ratios with 95% confidence intervals, and diamond shapes represent pooled estimates for each subgroup.
